# The Protein Tyrosine Phosphatase 1B Modulates the Activation of Yes-Associated Protein and Sensitizes to Cytotoxic Chemotherapy in Preclinical Models of Cholangiocarcinoma

**DOI:** 10.3390/cells14191560

**Published:** 2025-10-08

**Authors:** Ryan D. Watkins, Jennifer L. Tomlinson, EeeLN H. Buckarma, Hendrien Kuipers, Danielle M. Carlson, Nathan W. Werneburg, Daniel R. O’Brien, Chen Wang, Rory L. Smoot

**Affiliations:** 1 Department of Surgery, Mayo Clinic, Rochester, MN 55905, USA; 2 Division of Gastroenterology and Hepatology, Mayo Clinic, Rochester, MN 55905, USA; 3 Department of Quantitative Health Sciences, Mayo Clinic, Rochester, MN 55905, USA; 4 Department of Biochemistry and Molecular Biology, Mayo Clinic, Rochester, MN 55905, USA

**Keywords:** cisplatin, gemcitabine, hippo pathway, tyrosine phosphatase

## Abstract

**Highlights:**

- PTP1B is a YAP-interacting phosphatase that can decrease the tyrosine phosphorylation and transcriptional activity of YAP.
- PTP1B overexpression can decrease tumor growth *in vivo* and can increase sensitivity to standard of care chemotherapy in CCA.

**Abstract:**

Lacking effective therapeutics, cholangiocarcinoma (CCA) remains a deadly malignancy of the biliary tract. The Hippo pathway effector protein Yes-associated protein (YAP) is implicated in CCA pathogenesis and chemotherapeutic resistance; however, the oncogenic mechanisms underlying YAP regulation remain incompletely understood. An enhanced understanding of YAP and its role in CCA may uncover novel therapeutic targets and better define resistance pathways. Human CCA cells and murine syngeneic CCA models were utilized to explore the molecular relationship of YAP and protein tyrosine phosphatase 1B (PTP1B). Previous work in CCA has demonstrated that YAP interacts with multiple protein tyrosine phosphatases, including SHP2 and PTP1B. We observed that PTP1B pharmacologic inhibition was associated with increased cell proliferation and YAP target gene expression, while genetically enforced overexpression of PTP1B was associated with a decrease in YAP activation. Treatment of CCA cells *in vitro* and syngeneic, orthotopically implanted CCA murine tumors *in vivo* with standard cytotoxic chemotherapy, gemcitabine/cisplatin, had enhanced efficacy in the setting of PTP1B overexpression. These findings demonstrate that pYAP^Y357^ can be modulated through protein tyrosine 1B phosphatase activity, and reducing pYAP^Y357^ through enhanced phosphatase levels can sensitize CCA to chemotherapy.

## 1. Introduction

Cholangiocarcinoma (CCA) is a cancer arising from the biliary epithelium with an increasing incidence and limited overall survival [1,2,3,4]. CCA tumors are classified by anatomic site of origin as intrahepatic CCA (iCCA), perihilar CCA (pCCA), and distal CCA (dCCA) [1,2,3,4]. Currently approved treatments—including cytotoxic chemotherapy, targeted therapies, and immunotherapy—have had only modest effects on patient outcomes [4,5,6,7,8,9]. While the combination of gemcitabine and cisplatin has been the backbone of therapy for greater than a decade, response rates are only 25–30% with an overall survival of less than 1 year in patients with advanced or metastatic disease [9]. The addition of immunotherapy to gemcitabine and cisplatin has been recently approved based on positive findings from two large randomized, phase III trials [6,7]. However, the addition of immunotherapy has not resulted in clinically meaningful improvement for most patients. The genomic landscape of CCA is highly variable and greatly influenced by etiology [10,11,12]. However, some commonalities exist which provide an opportunity to employ targeted therapeutics for small subsets of patients. Key emerging therapeutic targets in cholangiocarcinoma encompass FGFR, IDH, the RAS–RAF–MEK–ERK signaling cascade, HER2 amplification, DNA mismatch repair deficiency, and NTRK fusions [13,14,15]. However, response rates for targeted therapy are also low and treatments provide only a short duration of efficacy [5,8,16,17,18,19]. Overall, therapeutic resistance is common in CCA and understanding mechanisms regulating cancer cell growth and resistance to therapy remains paramount to improving the survival for these patients [20].

The transcriptional co-activator Yes-associated protein (YAP) is activated in CCA and is a known mediator of therapeutic resistance [21]. YAP activity is regulated by a cascade of serine/threonine kinases called the Hippo pathway. When activated, the Hippo pathway culminates in YAP serine phosphorylation and subsequent cytosolic sequestration. Prevented from interacting with transcription factors, activation of the Hippo pathway acts as a brake on YAP transcriptional activity [22]. In contrast to the inhibitory regulation of the Hippo pathway on YAP, we have previously demonstrated a role for tyrosine phosphorylation as an activating mark of YAP that promotes nuclear localization and target gene expression [23,24,25,26]. Interestingly, the majority of human CCA tumors demonstrate aberrant nuclear-localized YAP [27]. In mice, CCA arises following genetic activation of YAP or deletion of components of the Hippo pathway [28]. In our previous work, we demonstrated that YAP interacts with multiple protein tyrosine phosphatases, including SHP2 and PTP1B, in CCA [23]. Evaluation of SHP2 signaling in CCA models demonstrated that SHP2 could reduce YAP activation by reducing tyrosine phosphorylation. Accordingly, inhibition of SHP2 resulted in increased YAP activity and chemotherapeutic resistance. These findings suggest a role for tyrosine phosphatases in the regulation of YAP activity and response to chemotherapy in CCA.

PTP1B is a protein tyrosine phosphatase associated with insulin resistance that has demonstrated both tumor suppressive and tumor promoting functions, depending on the biologic context [29]. Tumor suppressive functions of PTP1B were observed secondary to downregulation of receptor tyrosine kinase (RTK) and BCR-ABL signaling along with decreased activation of Src kinases [30]. In contrast, when PTP1B activity is suppressed, it has been associated with more aggressive tumor behavior and therapeutic resistance in breast and pancreas cancer [31]. Clinically, inhibition of PTP1B has been undertaken in early phase trials for the treatment of diabetes. PTP1B inhibition was also tested in a phase I clinical trial in breast cancer due to its effect on HER2 signaling [32]. The trial outcomes, however, were not reported and the trial is no longer recruiting. The role of PTP1B in cholangiocarcinoma and its subsequent effect on oncogenic signaling has not been explored.

Herein, we hypothesized that overexpression of PTP1B in CCA would restrain YAP activity, attenuate tumor cell proliferation, and sensitize CCA cells to chemotherapy. In these studies, we observed that PTP1B inhibition was associated with increased cell proliferation and YAP target gene expression, while overexpression of PTP1B was associated with a decrease in YAP activation. Furthermore, we demonstrated that upregulation of PTP1B levels lead to decreased tumor growth in mice that had undergone orthotopic implantation of syngeneic murine CCA cells; improved response to cytotoxic chemotherapy in preclinical models of cholangiocarcinoma was also observed.

## 2. Materials and Methods

### 2.1. Cell Culture

The human cholangiocarcinoma cell lines HuCCT1 and KMCH and murine CCA cell lines SB1 and FAC were maintained under conditions previously described [23]. Mycoplasma contamination testing was utilized with PlasmoTest^TM^-Mycoplasma Detection kit (InvivoGen, San Diego, CA, USA). SB1 and FAC cells were generated through biliary instillation and previously characterized [21,33].

### 2.2. Antibodies and Reagents

TCS401 (Selleck Chemicals, Houston, TX, USA), doxycycline hyclate (Sigma-Aldrich, Inc., St. Louis, MO, USA) and gemcitabine and cisplatin (Cayman Chemical, Ann Arbor, MI, USA) were added to cells at final concentrations from 0.05 to 100 µM per experimental design. The following primary antibodies were used for immunoblot analysis: actin (Santa Cruz, Dallas, TX, USA), YAP^Y357^ (ab62751 abcam, Cambridge, MA, USA), total YAP (63.7 Santa Cruz, Dallas, TX, USA; 4912 Cell Signaling Technology, Danvers, MA, USA), and PTP1B (Cell Signaling Technology, Danvers, MA, USA). The primary monoclonal antibodies used for immunofluorescence and/or immunohistochemistry included total YAP (63.7 Santa Cruz, Dallas, TX, USA), phospho YAP^Y357^ (ab62751 abcam, Cambridge, MA, USA), and BrdU (Rockland Immunochemicals, Inc., Limerick, PA, USA). ProLong Antifade with 4′,6-diamidino-2-phenylindole (DAPI, Thermo Fisher Scientific, Waltham, MA, USA) was used for nuclear staining.

### 2.3. Immunoblot Analyses and Immunofluorescence

Studies were performed as previously described [23].

### 2.4. Quantitative RT-PCR

Studies were performed as previously described [23]. Technical replicates were completed for each run and a minimum of three biologic replicates completed for each condition/cell line. The primer sequences 5′ to 3′ are listed below:
**Gene****Forward Primer Sequence (5′ to 3′)****Reverse Primer Sequence (5′ to 3′)**Human *CYR61*GAGTGGGTCTGTGACGAGGATGGTTGTATAGGATGCGAGGCTHuman *NUAK2*GATGCACATACGGAGGGAGATTATCACGATCTTGCTGCTGTTCTMouse *Ctgf*CACTCTGCCAGTGGAGTTCAAAGATGTCATTGTCCCCAGG*18 s*CGCTTCCTTACCTGGTTGATGAGCGACCAAAGGAACCATAHuman *GAPDH*TCAAGGCTGAGAACGGGAAGCGCCCCACTTGATTTTGGAG

### 2.5. PTP1B Overexpression

The coding region of PTP1B was cloned using high fidelity Taq polymerase (Thermo Fisher Scientific, Waltham, MA, USA) and ligated into a doxycycline inducible vector, pAK_Tol2_TRE_Puro, a gift from Michael Yaffe (Addgene plasmid # 130259, Addgene, Watertown, MA, USA). PTP1B expression was induced by administration of doxycycline (15 µg/mL) in culture medium. Cells were transfected by electroporation with empty control or the PTP1B vector and underwent puromycin selection to obtain stable cell lines.

### 2.6. Cell Viability

CCA cell lines were plated in triplicate in 96 well plates and allowed to grow for 24 h before drug treatment. Cell viability was assessed using CellTiter-Glo^®^ Luminescent Cell Viability Assay according to the manufacturer’s recommendations (Promega, Madison, WI, USA).

### 2.7. In Vivo Efficacy Studies

The study protocol was approved by the institutional animal care and use committee at Mayo Clinic. Experiments were carried out using 8-week-old male C57BL/6J mice (Jackson Laboratory, Bar Harbor, ME, USA). Mice were housed 5 per cage under standard conditions (12 h light/dark cycle, controlled temperature and humidity) with ad libitum access to food, water, and environmental enrichment throughout the experiment. Mice were monitored daily for health and behavior. Humane endpoints included weight loss > 20%, inability to access food/water, impaired mobility, or signs of distress, at which point animals were humanely euthanized in compliance with IACUC protocols.

Two experimental groups were established using SB1 and FAC cells. Each experimental group was subdivided into four treatment arms: (1) empty vector with intraperitoneal saline, (2) PTP1B overexpression vector with intraperitoneal saline, (3) empty vector with gemcitabine/cisplatin, and (4) PTP1B overexpression vector with gemcitabine/cisplatin. The empty vector plus saline and empty vector plus gemcitabine/cisplatin subgroups served as the vehicle controls. Each mouse was one experimental unit; 24 and 26 mice were used in the SB1 and FAC arms, respectively, with 4–9 mice per treatment group. Sample size was based on previous work utilizing SB1 and FAC cell lines. Mice were anesthetized with isoflurane. A laparotomy was performed and 1 × 10^6^ cells resuspended in 100 µL of DMEM were injected in the liver. Analgesia was provided using local anesthetic administered pre-operatively and once daily for two consecutive days post-operatively, consistent with IACUC-approved protocols. Pre-established exclusion criteria included mice that expired or required euthanasia prior to study endpoint or failed to form tumors due to poor injection or reduced tumor cell viability. No animals met exclusion criteria, and no unexpected mortality occurred. All mice were included in the final analysis.

Randomization was performed using a computer-generated sequence. Allocation was concealed from personnel performing euthanasia and data collection. Mice were not housed by treatment arm or subgroup. Treatment order of cages and mice within each cage was randomized. Mice were given doxycycline chow for enforced plasmid expression. Drug treatment was initiated 2–3 weeks after tumor implantation. Combination gemcitabine (4 mg/kg) and cisplatin (1 mg/kg) or normal saline was delivered by intraperitoneal injection for 2 weeks (on days 0, 3, 6, and 9). Administration of gemcitabine was 11 h after light onset (HALO) followed by cisplatin 15 HALO [34]. Animals were euthanized and tumors extracted 14 days after the first treatment administration. Extracted tumor weights were collected as the primary outcome measure.

Statistical analyses were performed using GraphPad Prism, version 10.3.1. Pairwise comparisons between treatment arms were conducted using the Mann–Whitney U test, chosen due to small sample sizes and non-normal data distribution. All tests were two-tailed, with *p* < 0.05 considered statistically significant.

## 3. Results

### 3.1. PTP1B Interacts with YAP and Is Inversely Correlated with YAP Nuclear Localization

**Figure 1:**
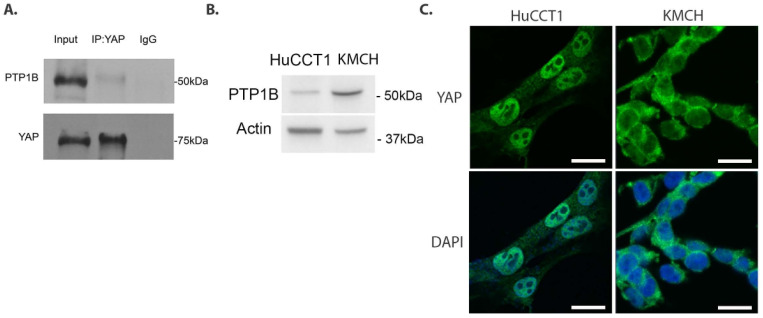
YAP and PTP1B interact. (**A**) Immunoblot analysis of PTP1B following immunoprecipitation of YAP in HuCCT1. (**B**) Immunoblot analysis of baseline PTP1B expression in human CCA cell lines, *in vitro*. Molecular (**C**) YAP confocal immunofluorescence with nuclear DAPI, 40× magnification, scale bars: 50 µm. All panels represent n ≥ 2 independent experiments.

We previously identified PTP1B as a YAP-interacting molecule in an immunoprecipitation-mass spectrometry screen using a murine CCA cell line [23]. To validate the mass spectrometry screen, we performed co-immunoprecipitation for YAP and PTP1B in HuCCT1 cells, which confirmed a YAP–PTP1B interaction (Figure 1A). Given the interaction between YAP and PTP1B, we investigated whether this interaction could result in a functional change in YAP status using two human CCA cell lines, KMCH and HuCCT1. These cell lines have contrasting YAP activation states along with differing expressions of SHP2, another YAP-interacting phosphatase [23]. Specifically, KMCH cells have demonstrated lower YAP activation status and higher SHP2 expression as compared to the HuCCT1 cell line. We similarly compared the PTP1B levels by immunoblot and YAP localization by immunofluorescence in these two cell lines (Figure 1B,C). With respect to YAP localization in HuCCT1 and KMCH cell lines, our observation is supported by prior work [23]. PTP1B levels were higher in the KMCH cell line which was accompanied by decreased nuclear-localized YAP. These data are consistent with a model where PTP1B could be regulating YAP subcellular localization by altering the tyrosine phosphorylation status.

### 3.2. PTP1B Inhibition Activates YAP in Cholangiocarcinoma Cells

**Figure 2:**
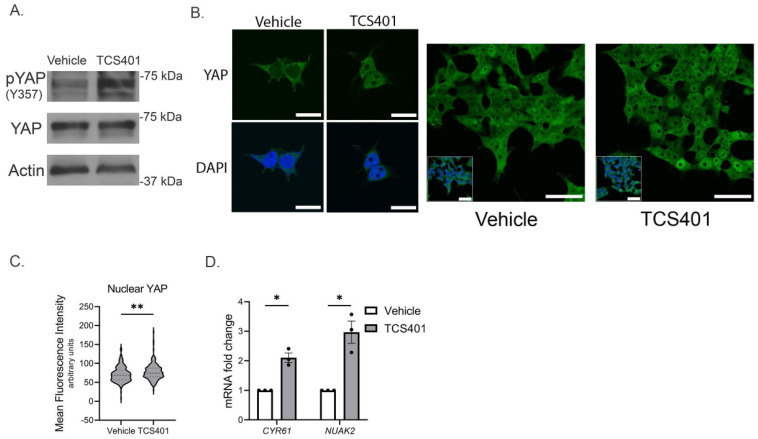
PTP1B inhibition increases YAP activity via tyrosine phosphorylation. (**A**) Immunoblot analysis of YAP tyrosine (357) phosphorylation following KMCH cell exposure to vehicle (DMSO) or TCS401, 10 µM for 24 h. (**B**,**C**) YAP confocal immunofluorescence with quantification of nuclear YAP in KMCH cells exposed to vehicle or TCS401, 40× magnification, scale bars: 50 µm, including inset image (dashed line, median; dotted lines, upper or lower quartiles). (**D**) YAP target gene quantification by qRT-PCR normalized to vehicle in KMCH following exposure to TCS401 (10 µM) or vehicle for 24 h. All panels represent n ≥ 3 biologic replicate experiments, significance was determined by unpaired two-tailed Student’s *t*-test: * *p* < 0.05, ** *p* < 0.01.

To directly evaluate the effects of PTP1B on YAP activation, we first examined the functional implications of the PTP1B–YAP interaction via pharmacologic PTP1B inhibition. We performed this line of experimentation in KMCH cells with higher levels of PTP1B expression in order to better demonstrate the effects of PTP1B inhibition. We exposed the KMCH cell line to TCS401, a PTP1B inhibitor. PTP1B inhibition was associated with an increase in YAP^Y357^ phosphorylation (Figure 2A). Consistent with our previous work that YAP^Y357^ phosphorylation is an activation mark for YAP, we also noted redistribution of YAP from the cytoplasm to the nucleus (Figure 2B,C) which resulted in enhanced expression of YAP target genes, *CYR61* and *NUAK2* (Figure 2D). These data suggest that PTP1B negatively regulates the co-transcriptional activity of YAP through modulation of YAP^Y357^ phosphorylation status.

### 3.3. PTP1B Overexpression Decreases YAP Activation and Cellular Proliferation

**Figure 3:**
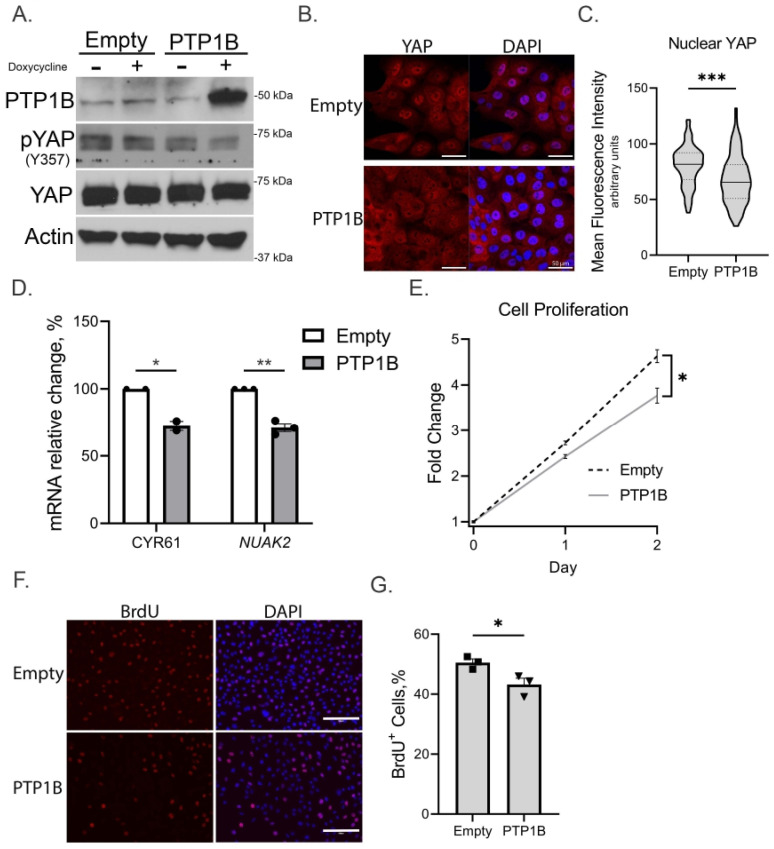
Increased PTP1B expression inhibits YAP and cellular proliferation in human CCA cells *in vitro*. HuCCT1 cells expressing empty control vector or PTP1B vector at baseline and following exposure to doxycycline. (**A**) Immunoblot evaluation of PTP1B expression and YAP activation. (**B**,**C**) Immunofluorescence microscopy of YAP subcellular localization and nuclear YAP quantification, 40× magnification, scale bars: 50 µm (solid line, median; dotted lines, upper or lower quartiles). (**D**) qRT-PCR evaluation of YAP target genes CYR61 and NUAK2. (**E**) Cell growth kinetics obtained by measuring cell numbers at baseline and every 24 h. (**F**,**G**) Immunofluorescence for BrdU cells with quantification of % positive cells per replicate, 10× magnification, scale bars: 150 µm. All panels represent n ≥ 2 biologic replicate experiments, significance was determined by unpaired two-tailed Student’s *t*-test: * *p* < 0.05, ** *p* < 0.01, *** *p* < 0.001.

To further understand the role of PTP1B in regulation of YAP activity and CCA cell proliferation, we next evaluated the effects of enforced expression of PTP1B in multiple CCA cell lines. We utilized HuCCT1 and two mouse cell lines, SB1 and FAC. HuCCT1 was utilized in this line of experimentation given its lower expression of PTP1B; SB1 and FAC murine CCA cell lines were concurrently analyzed given the planned *in vivo* experimentation. A doxycycline-inducible PTP1B overexpression construct was employed and following exposure of the cells to doxycycline was associated with an increase in PTP1B (Figure 3A and Supplemental Figure S1A). In HuCCT1 cells, overexpression of PTP1B was associated with decreased YAP tyrosine phosphorylation (Figure 3A), redistribution of YAP to the cytoplasm (Figure 3B,C), and reduction in YAP target gene expression *CYR61* and *NUAK2* (Figure 3D). These changes in YAP signaling were associated with reduction in cellular proliferation and DNA synthesis (Figure 3F,G).

**Figure 4:**
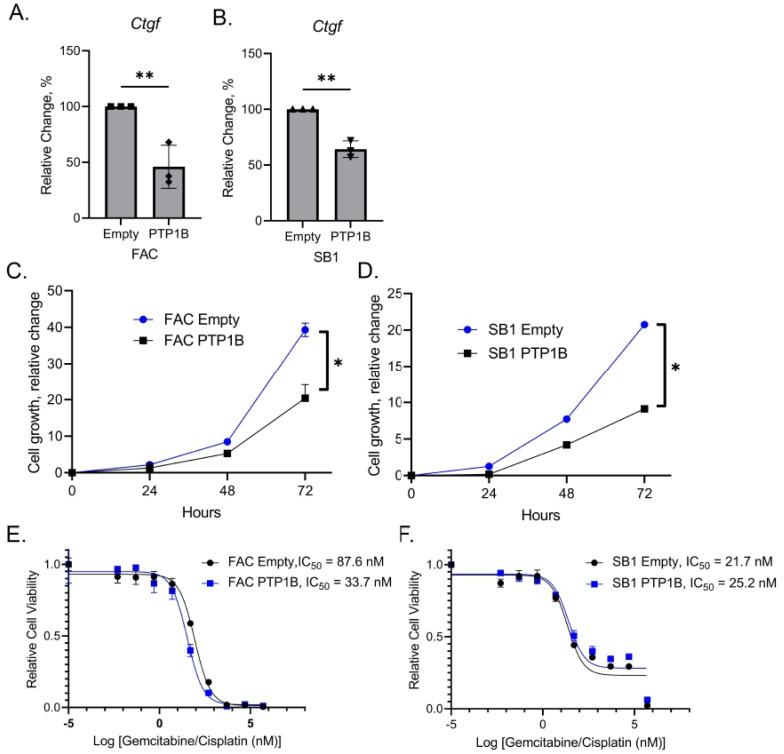
Increased PTP1B expression inhibits YAP and cellular proliferation in murine CCA cells *in vitro*. FAC and SB1 cells expressing the empty control vector or PTP1B expression vector were exposed to doxycycline and then evaluated by (**A**,**B**) qRT-PCR for YAP target gene *Ctgf* and (**C**,**D**) cell growth kinetics through cell counts at baseline and every 24 h. (**E**,**F**) FAC and SB1 cells were then exposed to doxycycline in combination with gemcitabine and cisplatin at increasing concentrations to identify the half maximal inhibitory concentration (IC_50_). FAC IC_50_ was significantly decreased when PTP1B was overexpressed (*p* < 0.01) but SB1 was not (*p* > 0.05). All panels represent n ≥ 3 biologic replicate experiments, significance was determined by unpaired two-tailed Student’s *t*-test: * *p* < 0.05, ** *p* < 0.01.

Similarly, induced expression of PTP1B in the murine CCA cell lines FAC and SB1 was associated with a decrease in levels of the YAP target gene *Ctgf* (Figure 4A,B) and decreased proliferation (Figure 4C,D). We next evaluated whether PTP1B overexpression sensitized murine CCA cells to the combination of gemcitabine and cisplatin. Cell viability after exposure to gemcitabine and cisplatin was reduced in FAC with enforced PTP1B expression as compared to controls, but not in SB1 cells (Figure 4E,F). These data suggest that increasing PTP1B levels in CCA cells decreases YAP activation, reduces cellular proliferation, and sensitizes some murine CCA cells to cytotoxic chemotherapy.

### 3.4. Overexpression of PTP1B Reduces Tumor Growth and Improves Response to Gemcitabine/Cisplatin in Syngeneic Murine CCA Models

**Figure 5:**
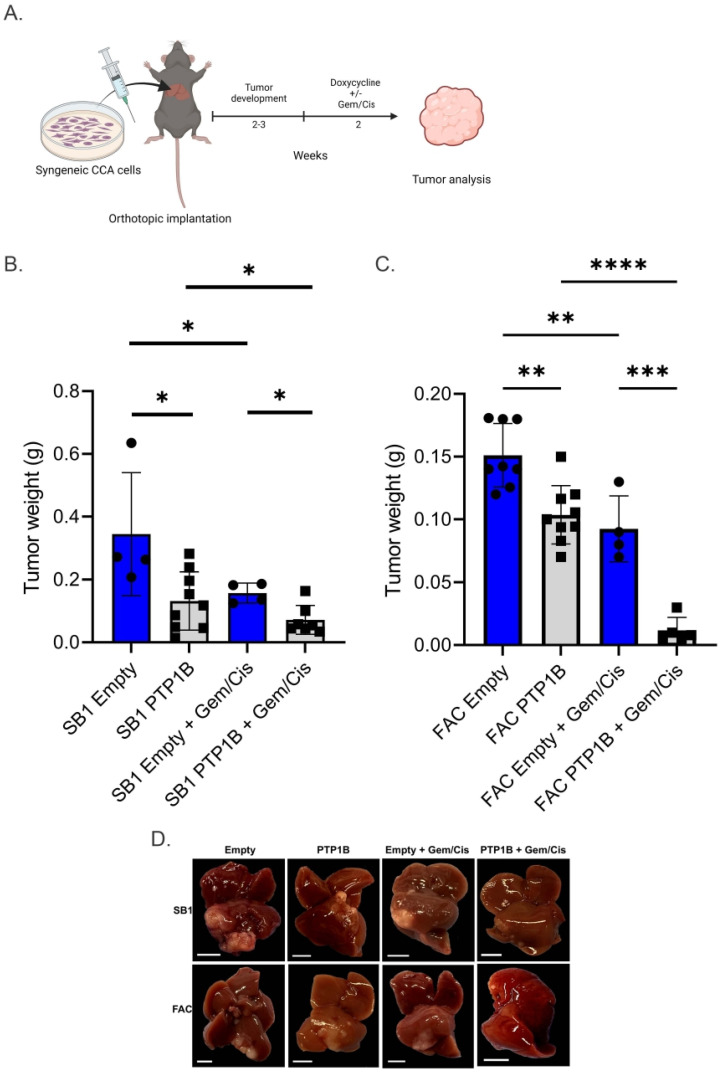
Increased PTP1B expression inhibits tumor growth and enhances chemotherapy sensitivity *in vivo*. (**A**) Experimental overview and timeline following SB1 and FAC CCA cell implantation, carrying either empty vector or PTP1B vector, and treatment with doxycycline chow with or without gemcitabine/cisplatin treatment. (**B**,**C**) SB1 and FAC tumor weights from mice treated with vehicle or gemcitabine/cisplatin with decreased tumor size in PTP1B expressing tumors which further enhanced sensitivity to gemcitabine/cisplatin treatment. (**D**) Gross images of murine livers containing SB1 or FAC tumors expressing empty control or PTP1B overexpression vectors +/− treatment with gemcitabine and cisplatin (Gem/Cis); scale bars 0.5 cm. Each data point on bar graphs represents an individual animal. Significance was determined by Mann–Whitney U test for each comparison and is denoted as * *p* < 0.05, ** *p* < 0.01, *** *p* < 0.001, **** *p* < 0.0001.

Finally, we evaluated the effects of PTP1B overexpression on *in vivo* growth of CCA tumors and sensitivity to the combination of gemcitabine and cisplatin in two syngeneic orthotopic murine models. SB1 or FAC CCA cells with and without enforced PTP1B expression were orthotopically implanted into the livers of C57BL6/J mice. Mice were administered doxycycline chow with or without combinatorial gemcitabine and cisplatin therapy (Figure 5A). Overexpression of PTP1B was associated with smaller tumors as compared to the tumor cells containing an empty control vector (Figure 5B,C). Furthermore, exposure to combination gemcitabine and cisplatin was associated with reduced tumor size as compared to control tumors (Figure 5B,C). These findings demonstrate that mice bearing tumors with increased PTP1B overexpression have decreased tumor growth and enhanced sensitivity to cytotoxic chemotherapy in syngeneic murine models.

## 4. Discussion

This study supports a role for tyrosine phosphatase PTP1B in restraining YAP activity and tumor growth in cholangiocarcinoma. These data indicate that (1) protein tyrosine phosphatase 1B can modulate the tyrosine phosphorylation status of YAP, (2) there is an inverse relationship between PTP1B activity and YAP activation and cellular proliferation, and (3) PTP1B activity can modulate response to standard of care chemotherapy *in vivo*.

This work provides insight into YAP regulation and oncogenesis beyond that of the canonical Hippo pathway and furthers our understanding of YAP biology. Our cumulative observations further support a model of YAP activation through tyrosine phosphorylation resulting in nuclear retention and function; they also suggest a role for protein tyrosine phosphatase 1B activity, whether via direct dephosphorylation or indirectly through modulation of other pathway phosphatases, in restraining YAP signaling. In addition to PTP1B, we have previously described the function of SHP2, a tyrosine phosphatase, which acts similarly to restrain YAP activation in cholangiocarcinoma via tyrosine modification [23]. In combination with our previous results, this work highlights the complex nature of YAP signaling in cholangiocarcinoma, as both PTP1B and SHP2 seem to work in similar manners and further illustrates the role of tyrosine phosphatases in YAP signaling. This overlap in YAP signaling control may be one of the reasons that cholangiocarcinoma has been characterized as an aggressive tumor that tends to respond poorly to chemotherapeutic agents and can rapidly develop therapy resistance. These observations provide further our understanding of YAP regulation in CCA and our ability to therapeutically target the YAP pathway and enhance current standard of care cytotoxic regimens.

Tyrosine phosphatases are frequently mutated in CCA and can function either as tumor suppressor genes or, less commonly, as oncogenes [35,36]. While it is difficult to target phosphatases when they function as tumor suppressor genes, like PTP1B, the objective has merit given their ability to modulate cholangiocarcinoma sensitivity to standard cytotoxic chemotherapeutics. Targeting pathways where there is a downregulated inhibitory function, like PTP1B, may become easier as gene therapeutics advance. This proposition will require additional study on the mechanisms by which PTP1B is regulated in order to develop effective therapeutic strategies.

PTP1B transcriptional expression is controlled by various mechanisms including metabolic stress and Y-box binding protein-1 (YB-1). YB-1 binds to a promoter region of PTP1B and enhances its expression [37]. Interestingly, YB-1 has been shown to be upregulated, promote stemness, and drive cisplatin insensitivity in cholangiocarcinoma through an AKT/B-catenin pathway [38]. These observations, combined with our findings, support the need to further understand the regulatory mechanisms exerted by PTP1B in cholangiocarcinoma. While outside the scope of this manuscript, these observations outline important future work to develop targeting strategies.

Our work highlights the ability of PTP1B to interact with and modulate YAP in cholangiocarcinoma, which has not been previously described. As a phosphatase, PTP1B, has many downstream targets, sometimes tissue specific, with complex regulatory pathways. Accordingly, while the PTP1B–YAP interaction was our focus and reduced YAP activity has been shown to correlate with enhanced chemosensitivity, we cannot ascribe the observed phenotype solely to decreased YAP activity. Additionally, while PTP1B activation may be beneficial in the treatment of CCA, systemic activation could have undesirable and complex off-target effects; therefore, tumor-specific targeting may be necessary. Thus, investigating other PTP1B substrates or methods for tissue-specific PTP1B targeting could be important areas for future research.

A limitation of this study is that experiments were performed in a subset of available CCA cell lines. Future studies will be needed to validate these findings across a broader panel of models to ensure generalizability. Additionally, we observed that SB1 cells, with constitutively active YAP, were resistant to cytotoxic chemosensitizion with concurrent PTP1B overexpression *in vitro*; this pattern was not maintained *in vivo* [21]. One explanation for this observation is that the tumor microenvironment modifies YAP signaling dependencies *in vivo*, rendering SB1 tumors more responsive to PTP1B modulation. This aspect of the differential treatment effects in varying environments is outside the scope of this paper and thus not fully explored. However, it remains an important question for future investigation.

## 5. Conclusions

Overall, our study further supports the role of YAP tyrosine phosphorylation in cholangiocarcinoma growth and chemoresistance with PTP1B being a novel regulator of YAP tyrosine signaling. The PTP1B-YAP signaling pathway could represent an area for future targeted therapy development as our understanding of the role of YAP in chemotherapy resistance continues to grow.

## Data Availability

The data that support the findings of this study are available from the corresponding author upon reasonable request.

## Supplementary Materials

The following supporting information can be downloaded at: https://www.mdpi.com/article/10.3390/cells14191560/s1; Figure S1: Murine CCA cell expression of PTP1B.

## Abbreviations

The following abbreviations are used in this manuscript:

CCA	Cholangiocarcinoma
ECL	Enhanced chemiluminescence
HALO	Hours after light onset
PTP1B	Protein tyrosine phosphatase 1B
YAP	Yes-associated protein
YB-1	Y-box binding protein
