# Supplementary material for: The Protein Tyrosine Phosphatase 1B Modulates the Activation of Yes-Associated Protein and Sensitizes to Cytotoxic Chemotherapy in Preclinical Models of Cholangiocarcinoma
Source: Cells. 2025 Oct 8;14(19):1560. doi: 10.3390/cells14191560 (PMC12523963; doi:10.3390/cells14191560)
Supplement: Supplementary file 1 [file cells-14-01560-s001.zip › cells-3888815-supplementary.pdf]

The Protein Tyrosine Phosphatase 1B Modulates the Activation of Yes-Associated Protein and Sensitizes to Cytotoxic Chemotherapy in Preclinical Models of Cholangiocarcinoma

Ryan D. Watkins[1#], Jennifer L. Tomlinson[1#], EeeLN H. Buckarma [1], Hendrien Kuipers[2], Danielle M. Carlson[1], Nathan W. Werneburg[2], Daniel O'Brien[3], Chen Wang[3], Rory L. Smoot[1,4*]

Supplementary Material

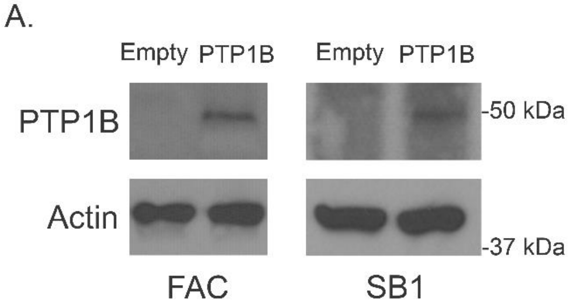

Supplementary Figure S1. Murine CCA cell expression of PTP1B. A) FAC and SB1 cells carrying the empty control vector or PTP1B vector following doxycycline exposure immunoblot analysis for PTP1B.
